# Translating the consent form is the tip of the iceberg: using cognitive interviews to assess the barriers to informed consent in South African health facilities

**DOI:** 10.1080/26410397.2024.2302553

**Published:** 2024-01-26

**Authors:** Nirvana Pillay, Nobukhosi Ncube, Kearabetswe Moopelo, Gaolatlhe Mothoagae, Olivia Welte, Manape Shogole, Nasiphi Gwiji, Lesley Scott, Noma Moshani, Nicki Tiffin, Andrew Boulle, Frances Griffiths, Lee Fairlie, Ushma Mehta, Amnesty LeFevre, Kerry Scott

**Affiliations:** a Senior Lecturer, Department of Sociology, University of the Witwatersrand, Johannesburg, South Africa; Director, Sarraounia Public Health Trust, 20 4th Avenue, Parktown North, Johannesburg, 2193, South Africa. *Correspondence:* Nirvana.Pillay@wits.ac.za; b Social Scientist, Sarraounia Public Health Trust, Johannesburg, South Africa; c Social Scientist, Sarraounia Public Health Trust, Johannesburg, South Africa; d Social Scientist, Sarraounia Public Health Trust, Johannesburg, South Africa; e Social Scientist, School of Public Health and Family Medicine, University of Cape Town, Cape Town, South Africa; f Social Scientist, Sarraounia Public Health Trust, Johannesburg, South Africa; g Social Scientist, School of Public Health and Family Medicine, University of Cape Town, Cape Town, South Africa; h School of Public Health, University of Cape Town, Cape Town, South Africa; i Social Scientist, School of Public Health, University of Cape Town, Cape Town, South Africa; j Professor, Life Sciences Building, South African Bioinformatics Institute, University of the Western Cape, Bellville, South Africa; k Professor, School of Public Health, University of Cape Town, Cape Town, South Africa; l Professor, Warwick Medical School, UK; Professor, Centre for Health Policy, School of Public Health, University of the Witwatersrand, Johannesburg, South Africa; m Director of Maternal and Child Health, Wits RHI, Faculty of Health Sciences, University of the Witwatersrand, Johannesburg, South Africa; n Associate Professor, School of Public Health, University of Cape Town, Cape Town, South Africa; o Associate Professor, School of Public Health, University of Cape Town, Cape Town, South Africa; p Independent research consultant, Toronto, Canada; Associate Faculty, Johns Hopkins School of Public Health, Baltimore, MD, USA

**Keywords:** informed consent, data use, language, maternal health, public health

## Abstract

The increasing digitisation of personal health data has led to an increase in the demand for onward health data. This study sought to develop local language scripts for use in public sector maternity clinics to capture informed consent for onward health data use. The script considered five possible health data uses: 1. Sending of general health information content via mobile phones; 2. Delivery of personalised health information via mobile phones; 3. Use of women’s anonymised health data; 4. Use of child’s anonymised health data; and 5. Use of data for recontact. Qualitative interviews (*n* = 54) were conducted among women attending maternity services in three public health facilities in Gauteng and Western Cape, South Africa. Using cognitive interviewing techniques, interviews sought to:(1) explore understanding of the consent script in five South African languages, (2) assess women’s understanding of what they were consenting to, and (3) improve the consent script. Multiple rounds of interviews were conducted, each followed by revisions to the consent script, until saturation was reached, and no additional cognitive failures identified. Cognitive failures were a result of: (1) words and phrases that did not translate easily in some languages, (2) cognitive mismatches that arose as a result of different world views and contexts, (3) linguistic gaps, and (4) asymmetrical power relations that influence how consent is understood and interpreted. Study activities resulted in the development of an informed consent script for onward health data use in five South African languages for use in maternity clinics.

## Plain Language Summary

In the wake of growing digitisation of personal health data, greater scrutiny is needed on the language of informed consent and the processes for soliciting consent in health care facilities. Qualitative interviews using cognitive interviewing techniques were used to develop and refine consent language in English, Sesotho, isiXhosa, isiZulu and Setswana for the onward use of health data among maternity clients in public sector primary health clinics in the Western Cape and Gauteng provinces of South Africa. We found that translation in local languages and addressing individual words and phrases was only one barrier to requesting informed consent. Other barriers were cognitive mismatches between the question intent and how women understood the question, linguistic gaps that were linked to language and identity, and power dynamics that affected how women understood the consent script. Emerging language scripts used “/” to present words in multiple languages; a reflection of the multi-linguistic nature of communities in this context.

## Résumé

La numérisation croissante des données personnelles de santé a entraîné une augmentation de la demande de données sanitaires en aval. Cette étude visait à élaborer des scripts en langue locale à utiliser dans les maternités du secteur public afin de recueillir le consentement éclairé pour l’emploi ultérieur des données de santé. Le script envisageait cinq utilisations possibles des données de santé: 1. envoi de contenus d’informations sanitaires générales au moyen de téléphones portables; 2. transmission d’informations sanitaires personnalisées au moyen de téléphones portables; 3. utilisation des données de santé anonymisées des femmes; 4. utilisation des données de santé anonymisées des enfants; et 5. utilisation des données en vue de recontacter les patientes. Des entretiens qualitatifs (*n* = 54) ont été menés auprès de femmes fréquentant les services de maternité dans trois établissements de santé publique du Gauteng et du Cap-Occidental, en Afrique du Sud. À l’aide de techniques d’entretien cognitif, les entretiens souhaitaient: (1) explorer la compréhension du script recueillant le consentement dans cinq langues sud-africaines, (2) évaluer la compréhension par les femmes de ce à quoi elles consentaient, et (3) améliorer le script sur le consentement. Plusieurs séries d’entretiens ont été réalisées, chacune suivie de révisions du script sur le consentement, jusqu’à ce que la saturation soit atteinte et qu’aucun échec cognitif supplémentaire ne soit identifié. Les échecs cognitifs étaient le résultat: (1) de mots et d’expressions qui ne se traduisaient pas facilement dans certaines langues, (2) d’inadéquations cognitives causées par différentes visions du monde et différents contextes, (3) de lacunes linguistiques, et (4) de rapports de pouvoir asymétriques qui influencent la manière dont le consentement est compris et interprété. Les activités de l’étude ont permis de mettre au point un script de recueil du consentement éclairé pour l’emploi ultérieur des données sanitaires dans cinq langues sud-africaines à utiliser dans les maternités.

## Resumen

La creciente digitalización de datos de salud personales ha causado un aumento de la demanda de datos de salud posterior. Este estudio buscaba elaborar un guion de terminología local para uso en centros de maternidad del sector público, con el fin de capturar consentimiento informado para el uso de datos de salud posterior. El guion consideró cinco posibles usos de datos de salud: 1. Envío de información de salud general vía teléfonos móviles; 2. Entrega de información de salud personalizada vía teléfonos móviles; 3. Uso de datos de salud de mujeres anonimizados; 4. Uso de datos de salud de niños anonimizados; y 5. Uso de datos para recontacto. Se realizaron entrevistas cualitativas (*n* = 54) con mujeres que asistieron a servicios de maternidad en tres establecimientos de salud pública en Gauteng y Cabo Occidental, Sudáfrica. Utilizando técnicas de entrevistas cognitivas, las entrevistas buscaban: (1) explorar la comprensión del guion de consentimiento en cinco idiomas sudafricanos, (2) evaluar la comprensión de las mujeres de lo que estaban consintiendo, y (3) mejorar el guion de consentimiento. Se realizaron múltiples rondas de entrevistas, cada una seguida de modificaciones al guion de consentimiento, hasta alcanzar saturación y no identificar fracasos cognitivos adicionales. Los fracasos cognitivos fueron el resultado de: (1) palabras y frases que no se traducían con facilidad a ciertos idiomas, (2) discordancias cognitivas que surgieron a causa de diferentes cosmovisiones y contextos, (3) brechas lingüísticas y (4) relaciones de poder asimétrico que influyen en las maneras de entender e interpretar el consentimiento. Las actividades del estudio produjeron un guion de consentimiento informado para el uso de datos de salud posterior en cinco idiomas sudafricanos para uso en centros de maternidad.

## Introduction

Definitions of informed consent have evolved over time but share a common focus on ensuring that the individual understands and freely agrees to an activity, which they sign permission for. In health care, informed consent is primarily obtained from patients for clinical care (e.g. laboratory tests, surgeries, medications), the use of their data to enhance care (e.g. using their phone number and clinical records to send them personalised health information, and for continuity of care through the provision of historical clinical data to clinicians), the use of their data for research (e.g. their anonymised clinical records analysed to assess the quality of care provided at the clinic and the conduct of observational research), and active participation in research (e.g. being randomised into a treatment trial or interviewed on their experiences). To be informed and free, consent processes must include full disclosure of the nature of the activity and the participant’s involvement, must convey this information in a manner the participant adequately comprehends, and must ensure the participant can freely choose whether to participate without compromising their access to quality care (p. 464).^1^

Informed consent is a central doctrine of clinical care and research, yet the extent to which consent forms are understood by healthcare clients and the extent to which consent processes are conducive to voluntary agreement has been a concern across varying contexts. A systematic review of the quality of informed consent across countries of different socio-economic contexts found that comprehension of study information, pressure to participate and understanding the right to withdraw from studies were some of the factors that affected the quality of consent across contexts.^2^ Less is known about the barriers to consent for onward use of data to enhance care or for research. However, informed consent studies are increasing in scope to include these issues as concerns about “big data” – as applied for modern analytics – become more tangible.^3,4^ The barriers to informed consent created by the need for translation between languages and cultures, and by power hierarchies between patients and the staff seeking consent, have rarely been explored in low- and middle-income countries and have not to date been explored in South Africa where this study takes place.

In this study, we developed informed consent scripts in five languages that asked women attending public sector maternity services whether or not they wanted to “opt-in” to types of health data use. In South Africa, the use of data is governed by three legislative acts: the National Health Act,^5^ the Promotion of Access to Information Act,^6^ and the Protection of Personal Information Act (POPIA)^7^ which was updated in 2021 making compliance more rigorous. This legislation requires that the South African public health care system, and all public and private institutions that collect and store non-anonymised data, ensure that their data collection, storage, use, and consent procedures align with POPIA rules. There is therefore a strong legislative framework, but in practice this is an evolving field that requires greater attention.

South Africa’s National Health Act, No.61 of 2003 states that: “The health care provider concerned must, where possible, inform the user as contemplated in subsection (1) in a language that the user understands and in a manner which takes into account the user's level of literacy.”^5^ Despite this mandate, consent language and procedures for requesting and documenting informed consent for clinical services *and* onward health data use have not been standardised within or across public sector health facilities in South Africa. The South African National Department of Health requested this research to expand the offering of the existing English-only maternal care informed consent script to other local languages, and to improve the content of the script to enhance women’s understanding of what they are consenting to. We translated the English consent script into 4 out of 11 of South Africa’s official languages and used cognitive interviews to refine the consent script to enhance understanding. In undertaking this process, we identified multiple barriers to informed consent extending beyond the words selected to the resonance of key concepts being conveyed, identifying a language of choice for consent processes, and power hierarchies in the health system. This study aims to present these barriers to informed consent and to provide insight into how they can be addressed or managed.

This work complements a companion paper^8^ which utilises in-depth interviews to determine user and provider perceptions of, and preferences for, the onward use of routine health data. Taken together, both papers aim to inform the improvement of informed consent procedures for the onward use of routinely collected health data collected as part of maternity care in South Africa. This is increasingly important to leverage valuable data (increasingly digitised) on women’s pregnancy journeys and birth outcomes in an ethical way to improve continuity and quality of care, health systems, and interventions targeted at improving the health of women and their new-borns.

## Methods

### Study context

This study on consent language was nested in the UBOMI BUHLE (Understanding Birth Outcomes from Mothers and Infants, Building Healthcare by Linking Exposures) project which has developed a pregnancy exposure registry (UBPER) across three provinces in South Africa. The consent language study sample was drawn from three midwife obstetric units: two in the Western Cape (urban and peri-urban) and one in Gauteng (urban) (Table 1).

**Table 1.** Brief contextual description of the study sites

Location	Western Cape urban midwife obstetric unit (MOU)	Western Cape peri-urban midwife obstetric unit (MOU)	Gauteng urban midwife obstetric unit (MOU)
Population density	15,000/km^2^	1700/km^2^	6357/km^2^
Socio-economic	40% of labour force unemployed; 71% average household income of R3200 (US$230) or less per month	14% of labour force unemployed; average household income: unknown	18.7% of households have no monthly income; 23.5% average household income of R19,600 (US$ 1250) or less per month
Social context	High levels of violence and poverty	Low crime rates	High crime rates
Built environment	Households: 48% considered as “informal” dwellings; 37% do not have a flush toilet; and 42% do not have piped water inside their dwelling/yard	Households: 12% considered as “informal” dwellings; 16% do not have a flush toilet; and 31% do not have piped water inside their dwelling/yard	Households: 15.8% considered as “informal” dwellings; 8,4% do not have a flush toilet; and 45% do not have piped water inside their dwelling/yard
Population groups	Black Africans (99%)	Coloured (72%)	Black Africans (99.7%)
Predominant language	isiXhosa	Afrikaans	isiZulu

Source: South African Census 2011^9^

### Research team

The research team consisted of nine researchers and three senior scientists. The researchers had a range of language competencies enabling us to translate, conduct, and analyse all interviews in the four local languages.

## Study methodology – cognitive interviews

Cognitive interviewing involves “the administration of draft survey questions while collecting additional verbal information about the survey responses, which is used to evaluate the quality of the response or to help determine whether the question is generating the information that its author intends”.^10^ We drew on an existing body of work that used cognitive interviews to qualitatively assess respondents’ understanding of keywords and phrases used in survey questions.^11,12^ This methodology seeks to bridge linguistic, social, and cultural gaps between the researchers who develop surveys and the populations who complete them to improve the match between research intent and respondent interpretation.^13^

In this study, cognitive interviews were conducted with beneficiaries attending public midwife-run obstetric unit services and were used to develop and refine informed consent language for onward health data use in four languages. We first adapted an existing English language consent script intended for implementation by antenatal care health facility staff through stakeholder consultation. The English consent script was then translated into four South African languages (isiZulu, isiXhosa, Setswana, Sesotho); these were selected because they included predominant languages in the study sites. At the time of the study, the MomConnect program had been suspended and our use case was generalised to capture a wider swathe of probable use cases likely to be available to maternal and obstetric unit beneficiaries. We explored the feasibility of having a simple, easy-to-understand one-page form which could be implemented in the context of routine care in a health system characterised by limited resources.

The consent script (Table 2) asked women whether or not they wanted to opt into five scenarios of onward health data use. Scenario 1 involved the delivery of general health information messages to beneficiaries using their mobile phone number, and encapsulates the National Department of Health’s current national mobile health program MomConnect;^14,15^ limitations with this consent process are reported elsewhere.^16^ Scenario 2 involved tailored health information messages – for example, appointment reminders, a service which is available in select public sector facilities. In Scenario 3, beneficiary consent is sought for the use of anonymised data for health services research related to the mother; in Scenario 4, beneficiary consent is sought for the use of anonymised data for health services research related to the child. Scenario 5 aims to consent beneficiaries to be re-contacted for future research.

**Table 2.** Original consent script

*Thank you for visiting the clinic. As you may have experienced, we have collected information about you and the baby you will deliver. This includes your age, weight, health, medicines you take, and similar information about your baby. This information is routinely collected in order for the Department of Health to provide you and your baby with health care services. The Department of Health would also like to ask you if we can use this information for other purposes which I will describe to you now. You can choose whether you agree or not for each of these purposes. It is your decision, and your answers will not change the care you receive. For purposes you agree to, we may ask for some additional information including your contact details.*		
1. **Can the Department of Health send you general health information about pregnancy and child health via mobile phone through text or WhatsApp?** For example, you will receive MomConnect messages about pregnancy and child health for up to 1 year following the birth of your child.	**Yes**	**No**
2. **Can the Department of Health send you specific information about your pregnancy and your child’s health through SMS or WhatsApp?** For example, you may receive alerts and reminders about your clinic visits, test results, and medicines.	**Yes**	**No**
3. **Can your health information be used without your name or personal details?** This information will be used to understand and improve the health of mothers and children and the health services they receive.	**Yes**	**No**
4. **Can your child’s health information [up until their 5th birthday] be used without your name, your child’s name, or personal details?** This information will be used to better understand and improve the health of mothers and children and how health services are delivered to them.	**Yes**	**No**
5. **Can the Department of Health contact you by telephone in the future to invite you to join other research studies?** We will keep your phone number and contact you if there are other studies you could take part in. At that time, you can decide whether you wish to participate or not.	**Yes**	**No**

## Study sample and recruitment

We drew on recent work using cognitive interviews to assess Respectful Maternity Care in India^12,17^ to determine the sample size for cognitive testing consent scripts in each language. We aimed to test Version 1 of the consent script with six respondents, Version 2 with four respondents and Version 3 with two respondents. Table 3 reflects the final sample of cognitive interviews.

**Table 3.** Sample of cognitive interviews per language tested

	Version 1	Version 2	Version 3	Total per language
isiZulu	6	3	2	11
isiXhosa	6	3	2	11
Setswana	6	3	2	11
Sesotho	6	3	2	11
English	5	3	2	10
Total across five languages	29	15	10	54

Clinic-based data collectors supported recruitment at the three sites. Recruitment and data collection occurred simultaneously in September and October 2021. A total of 54 interviews were conducted (*N* = 54); 43 interviews were face-to-face (*n* = 43) and 11 were telephonic (*n* = 11).

Recruitment for both sites varied as a result of COVID-19 infections and related safety protocols in clinics during data collection. Women were purposively recruited based on the language they identified as their first language or the language they felt most comfortable speaking. We faced major limitations with recruiting English first language speakers as none of the clinics were attended by such populations. For recruitment of women to test the English consent script, we selected women who confirmed that they were comfortable speaking, reading and writing English.

## Data collection

A semi-structured cognitive interview guide was used to assess and understand women’s interpretation of the consent script. First, the researcher asked the consent question exactly as it was written and sought to elicit a response (yes/no), as if they were actually taking a patient’s consent. Then the researcher asked a series of qualitative probes to understand how the respondent interpreted the question and why they gave the response provided. For each question, women were asked: whether they would say yes or no; why they would say yes or no; how they understood the question; what specific words in the question meant; and how they thought other women might respond to the same question. Women were also asked how they understood words and phrases used in the preamble to the consent questions; these included “consent”, “we have collected information about you”, “information”, “routinely collected”, “the Department of Health”, “It is your decision” and “your answers will not change the care you receive”.

Beneficiaries were compensated for travel and time using electronic vouchers ranging in amount from ZAR150 (US $8.01) to ZAR200 (US $10.69) across sites depending on established precedence in each clinic. The total duration of in-person interviews was between 30 and 60 minutes and telephonic interviews 60–90 minutes. At this time, Gauteng province had seen the end of the third COVID-19 wave and the study team were allowed access to the clinics and conducted all interviews face-to-face in the antenatal care section of the midwife obstetric unit (MOU). At the beginning of the study high COVID-19 prevalence in the Western Cape did not allow for clinic visits; interviews with women in the Western Cape therefore began telephonically. Telephonic interviews were longer because of challenges including cell phone battery life, poor audibility, network connectivity, phone sharing with other members of a household and/or lack of private space for participants to speak on the phone. We noted interview fatigue with many women; this was so with telephonic interviews because women lost concentration throughout the interview and also during in-facility interviews where women had waited for long hours at the antenatal clinic before participating in the interview. Some women were intimidated or confused by the nuances between questions. We addressed this by asking questions slowly, repeating questions in different languages and using examples. We consistently assured women that they were not being tested and there were no right or wrong answers.

All interviews were audio recorded, translated into English and transcribed.

## Data analysis

Detailed analysis of each cognitive interview sought to reveal mismatches between the intent of the original questions and how respondents interpreted them. There were two levels of analysis. The first level occurred iteratively, with data collection during daily debriefs between researcher pairs who went through the consent script and discussed from field notes and observation how respondents understood individual words and phrases, cognitive mismatches and any other areas where the consent script needed amendment. Notes were entered into a Google spreadsheet organised by language (sheets), domain (rows) and participants (columns). After an average of five to six interviews using Version 1 of the consent script, researchers made the necessary changes to proceed to the next iteration; the same process was followed for Version 2 (three interviews) and Version 3 (two interviews). Researchers assessed levels of data saturation for each version during the daily debriefs.

The second level of analysis involved thematic analysis of transcripts uploaded into Dedoose™ to identify key themes across all domains, as well as emergent findings. In the process of translation and back-translation of transcripts to English for coding, we found that phrases in vernacular languages were sometimes interpreted differently by transcribers and researchers; we are mindful that some nuances within each language may have been missed in the process. We mitigated this by thoroughly reviewing and re-listening to all audio recordings and transcripts, resubmitting transcripts for correction, and assigning researchers from our team who were most fluent in each language to check the quality of these transcripts. Thereafter, all researchers were involved in the identification of codes; transcripts were first coded using the general study themes, followed by each question in the questionnaire and then finally with any other emergent themes from the data. We carefully considered what constituted a cognitive failure as we were mindful of interview fatigue. After coding, changes that were made to the consent scripts were reviewed, adapted where necessary and verified. For this paper, analysis focused on identifying barriers to informed consent beginning with women’s understanding of the words and concepts in the consent form, and expanding to considerations of linguistics, identity and power.

## Methodological reflection

For all five scenarios in the consent script women were asked how they thought “other women” may answer the question they were asked. Importantly, some women stated that they could not answer for other women. However, others used this as an opportunity to offer critical insights that differed from their own responses. We reflected that in this case, this question allowed women the space to offer contrary viewpoints, without fear of the perceived consequence of not answering in a way that they thought was expected of them.

## Ethical approval

Ethical approval was obtained from institutional review boards at the University of Cape Town, Faculty of Health Sciences Human Research Ethics Committee Ref: 841/2020 dated 24 May 2021 and at the University of Witwatersrand, Human Research Ethics Committee (Medical) Ref: 201126 dated 19 July 2021.

### Findings

The respondents were all pregnant women who ranged in age from 16 to 40, with an average age of 29 years. They ranged from having completed seven years of formal education to 16 years, with an average of 11.4 years of formal education. About one-third had not finished secondary school (fewer than 12 years of education); almost half had completed secondary school and done no further studies (12 years of education); and 17% had completed additional schooling beyond secondary. About two-thirds of the respondents were unemployed. The remaining respondents included five students as well as secretaries, cleaners, shop assistants, a quality assurance analyst, domestic workers, and seasonal and factory workers.

**Figure 1.**
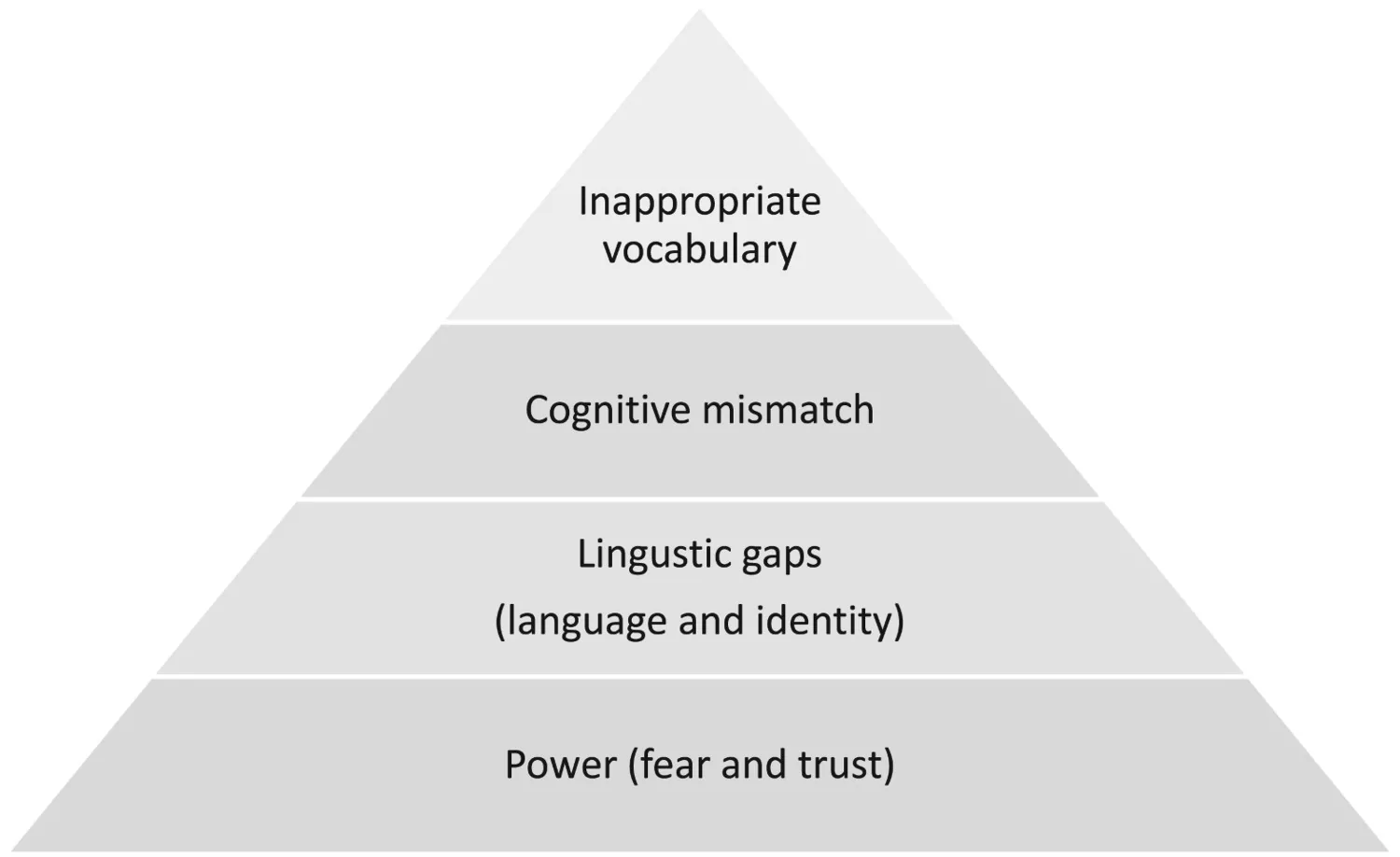
Barriers to informed consent

Figure 1 presents the four main barriers to informed consent. These barriers were the reasons why women said “yes” or “no” without really understanding what was being asked, therefore giving consent for things they may not really want or refusing consent for things that they may want.

The themes represent cross-cutting findings across all five languages. We highlight specific examples from all languages to describe challenges experienced with translation into local languages.

We drew on this analysis to finalise the consent script in each of the languages. The original and final version of the consent script in English is presented in Table 6. The final consent scripts in the four other languages are in Supplementary Tables 1–4.

#### Inappropriate vocabulary

Some words and phrases used in the original consent script were not well understood. Table 4 presents a summary of words/phrases identified and how we resolved these using select examples from different languages. The first issue related to the use of sub-optimal words in the first iteration of the consent script. This type of failure was relatively easy to address by substituting alternative, easier-to-understand words or by replacing the words with a synonym in addition to the original word, or by removing words that were redundant. One unique sub-issue relating to word choice occurred in English with the word “consent” which women were asked the meaning of even though it was not in the consent script. “Consent” was often misheard and repeated back as “concern”, understood to mean “have worry about something” (C_ANC_ENG_05) or “something that is always on your mind”. (C_ANC_ENG_06) Indeed, the two words sound like near-homonyms and researcher attempts to differentiate between them through pronunciation were unsuccessful. When researchers tried to differentiate between the two words by spelling out the word or defining “consent” it showcased a deeper conceptual blurring, wherein the “consent form” was also understood to showcase “concern” about the woman: “ … it’s like you are concerned to know things from me about the clinic so that’s what I understand”. (C_ANC_ENG_07) We then used “agree” or “permission” to avoid this auditory overlap and conceptual blurring.

**Table 4.** Vocabulary issues identified and how they were addressed

Issue	Original	Improved alternative
1. Original word selected was not understood, inappropriate or redundant; alternate or simpler word or phrase was used	**English: Preamble:** routinely	**English: Preamble:** every time
	**Sesotho: Preamble:** *tlhokomelo* [care]	**Sesotho: Preamble:** *Thuso* [help/assistance]
	**isiXhosa: Preamble:** *impilo* [health or life]	**isiXhosa: Preamble:** *isimo sempilo* [health status]
	**Sesotho: Preamble:** *le ditlhaiso-leseling tse* [this information]	**Sesotho: Preamble:** *le ditlhaiso-leseli/tsebo tse* [synonym for information i.e. knowledge added]
	**isiZulu**: **Q1:** *ngomakhalekhukhwini ngeSMS noma ngeWhatsApp* [via mobile phone using SMS or WhatsApp] **Sesotho: Q1**: *mohala ka molaetsa* [message via mobile phone]	**IsiZulu: Q1**: *ngeSMS noma ngeWhatsApp* [mobile phone – *makhalekhukhwini –* deleted because it is redundant when referring to SMS or WhatApp] **Sesotho: Q1:** *ka molaetsa* [mobile phone – *mohala* – deleted because it is redundant when referring to SMS or WhatApp]
	**Setswana***:* Q4: *di rebolwa* (are given or provided)	**Setswana: Q4:** *di neelwa* (are given or provided)
	**isiZulu: Q4:** *imininingwane yomuntu siqu* [personal information]	**isiZulu: Q4:** *imininingwane ekhombisa wena* [information that identifies you]
2. Word “routinely” not well understood; word repetition is used to emphasise the intended meaning	**Setswana: Preamble:** *kgapetsa* [routinely/frequently]	**Setswana: Preamble:** *kgapetsa-kgapetsa* [over and over again]
	**Sesotho: Preamble:** *khafetsa* [routinely/always]	**Sesotho: Preamble:** *ka mehla le mehla* [each and every time/always and always]
3. Difference in Q1 (general health information about pregnancy and child health) and Q2 (information about your pregnancy and your child’s health) was not seen as different; alternative or additional word/s for “information” used to differentiate Q1 from Q2	**English**: Q1. Can the Department of Health send you general health information about pregnancy and child health via mobile phone through text or WhatsApp? **English**: Q2. Can the Department of Health send you information about your pregnancy and your child’s health via your mobile phone through text or WhatsApp?	**English**: Q1. Can the Department of Health send you health information about pregnancy and child health through SMS or WhatsApp? This information will be general to all pregnant women and mothers. **English**: Q2. Can the Department of Health send you specific information about your pregnancy and your child’s health via your mobile phone through text SMS or WhatsApp?
	**isiZulu**: Q1: *imininingwane ejwayelekile yezempilo* [general health information] **isiZulu**: Q2: *imininingwane* *mayelana nokukhulelwa kwakho* [information about your pregnancy]	**isiZulu**: Q1: *ulwazi olujwayelekile mayelana nokukhulelwa kwabesifazane* [general knowledge about pregnancy] **isiZulu**: Q2: *ulwazi oluthile mayelana nokukhulelwa kwakho* [specific knowledge about your pregnancy]
	**isiXhosa**: Q1: *iinkcukacha zempilo ezithe gabalala* [general health information]	**isiXhosa**: Q1: *iinkcukacha / i-information zempilo ezithe gabalala/ezibanzi* [general/broad health information]

The second issue, in Sesotho and Setswana, was that a single word to convey the concepts “routinely” or “frequently” did not work, but worked when repeating the vernacular word which better expressed the idea of over and over again or each and every time. The third issue was differentiating between two questions that asked whether the Department of Health can send women: (1) “general health information about pregnancy and child health”, and (2) “information about your pregnancy and your child’s health”. Question 1 refers to general information that is available to all pregnant women and their children, and Question 2 refers to health information specific to the individual woman being consented and enrolled for ANC. This technical difference was not easily understood: “*they are the same … because they are all about getting information”* (ANC_ZUL_01) or “*They are the same because they both talk about the pregnancy and the child and messaging being sent via the phone”* (C_ANC_SOT_07). The final consent scripts were adapted to more accurately reflect the difference between the questions using emphasis, synonyms and for Q1 adding a sentence to explain more fully the meaning of the question.

#### Cognitive mismatches

A cognitive mismatch is a discrepancy between what is intended by the text and how the text is understood by participants.^11^ In cases where there was a cognitive mismatch, respondents reported that they found the question clear and provided confident answers, but when probed, their interpretation was very different from the intent of the question. We identified two questions (Questions 3 and 5) that failed to work as intended (cognitive mismatch) (Table 5).

**Table 5.** Cognitive mismatch between intent of the question and respondent’s interpretation

Question	Q3	Q5
**Original question**	**Can your health information be used without your name or personal details?** This information will be used to understand and improve the health of mothers and children and the health services they receive.	**Can the Department of Health contact you by telephone in the future to invite you to join other research studies?** We will keep your phone number and contact you if there are other studies you could take part in. At that time, you can decide whether you wish to participate or not.
**Intended meaning of question**	Anonymised data for health research (not for clinical care) to protect the patient’s identity	Permission to be recontacted. Potential future opportunities to participate in research studies
**Respondent interpretation**	• Anonymisation renders me invisible to health workers – my name is removed from health records and so my care will be compromised. “*When they are used without my name, it means that nothing will be remembered about me … ”* (ANC_XHO_11). “* … it is my personal details … So, you cannot use it without my name … It must be known that these are [participant’ name]” (ANC_TSW_03)* • Anonymisation makes it possible for others to scam me “*But, there is that mistrust, because some people, there is a lot of scams. You would think that people are helping you, just to find out that in actual fact they are deceiving you. So I’d prefer it if my information is kept confidential by the health facility and not used it in any other way.”* (ANC_ZUL_05) • Anonymisation is to shield people from shame and if I do not feel shame I do not need to be anonymous “*I cannot be anonymous because there’s nothing that I’m hiding … Because it’s something that is happening in the clinic mos. So, there is no need for someone to be anonymous.” (ANC_ENG_07)* • Anonymisation is identity theft because they are using my information without my name “*You are using my information without my name … And my personal … Details … No, I would feel abused, oh. My rights being abused”* (ANC_TSW_04). • I don’t understand how my information can be used to help others “*Why would they use my information to [for] the woman they will interview who [while] at the same time has [they have] her information? … How will my information help other women? I am confused, l don’t understand. How will it improve their lives?” (ANC_ZUL_08)*	• An opportunity to learn more [study] about my health i.e. a health education opportunity “*Because I love learning lots of things about my health and the baby’s.” (ANC_XHO_5)* “*I’d say yes because they’d still contact me via the phone for other studies and learn a thing or two myself in the process. Like they say you’re never too old to learn and I would learn more.” (ANC_SOT_05)* “* … maybe the one who would disagree is one who is not interested in knowing more about health … ” (ANC_ZUL_08)*
**Resolution**	Final version: “Can your health information be used anonymously (meaning: without your name or personal details) in health research? This information will be used by health researchers to understand and improve health services for mothers and children.” Where anonymity was conflated with not being known by health workers thereby compromising clinical care we added “*used anonymously (meaning: without your name or personal details) in health research”* to differentiate it from their clinical care where their identity would be known by those providing clinical care. Where anonymity was associated with being vulnerable to scams we added “in health research” and “This information will be used by health researchers … ” to explain the use of their data and minimise concerns about scams. We did not address the other mismatches as they were specific concerns raised by individual women and were not prevalent across the sample. This was similarly adapted in all languages – see Supplementary Tables 1-4.	Final version: “Can the Department of Health contact you by phone in the future to invite you to join other research? We will keep your phone number and contact you if there is other research you could take part in. At that time, you can decide whether you wish to participate or not.” We removed the word “study/ies” and used only “research” to emphasise that recontact may be for future research as “study” seemed to convey to women that it was an opportunity for them to study/learn more. Similarly adapted in all languages – see Supplementary Tables 1–4.

In the original script, the intention of Question 3 was to offer anonymity by having names removed from clinical records to convey to research participants that their data and information in health research would be protected. It was a cognitive mismatch because many respondents misinterpreted this form of anonymity to mean a range of things that differed from this intention (see Table 5). Most importantly, women often associate anonymity with being unknown to health workers and thereby having their clinical care compromised. A further concern was that it would be easier for others to use their personal information for scams. We addressed these particular cognitive mismatches by adding further explanatory text as seen in column 2 in Table 5.

The second cognitive mismatch was in relation to how women understood what it meant for them to be re-contacted to participate in future [research] studies. The researchers’ intent was for women to give permission to be re-contacted if there were future opportunities for them to participate in research studies as study participants. However, many women understood this to mean that they would have future opportunities to gain further health education i.e. to “study” more about their own health. We clarified this by removing the word study and using only “research”. We did not address this cognitive mismatch more deeply; for example, by explicitly stating that research is for wider benefit rather than for the specific research participants’ growth and education because it seemed unnecessarily complicated and overlooked the fact that research participants often do benefit from the process and gain information about health, such as through receiving a trial health education intervention.

These examples illustrate that cognitive mismatches are more than a misunderstanding of words, concepts, and phrases and are therefore more complex to resolve, often requiring expanding phrases to convey more meaning and using examples to support better understanding (see Table 5). They often result from different world views and social and cultural contexts and interpretations.

#### Linguistic gaps – language and identity

On invitation to participate in the study, women were asked to identify their first language or the language they were most comfortable reading, writing, and speaking. However, despite self-identifying as first-language speakers, women often found the consent script difficult to understand, and linguistic gaps were noted. There were two main, but related, reasons for this disconnect.

First, although women identified as speaking X as a first language they did not actually speak or understand it as well as Y [language]. There were complex reasons as to why they don’t consider Y to be their first language despite this anomaly. Language was often strongly associated with culture and place and not necessarily linguistic proficiency. For example, an isiZulu respondent reflected on the cultural importance of identifying as Zulu because her name was Zulu even though she wasn’t very comfortable speaking isiZulu – her name therefore conferred her identity. Another participant described her language of preference as “*my isiZulu*” (ANC_ZUL_05) highlighting her pride and ownership of her cultural identity. As discussed earlier, participants’ selected first language did not always reflect what they best understood linguistically, and they would often seek clarity in the language they better understood. For example, a self-identified first language isiXhosa speaker said:

*“Uhm … It was only difficult when I got here to give birth. The nurse was telling me to open my legs but used the **deep isiXhosa**. I asked her to repeat, they asked what language I speak, I said Setswana, and they explained that I must open my legs. There are some **deep Xhosa** words that I do not understand.”* (ANC_XHO_13)

Second, women often exist “between languages” and speak a mix of languages. This means that they don’t fit neatly into any one language category. For example, when women didn’t understand technical or complex words in one language they drew from other (sometimes multiple) languages to express themselves in response to the questions asked by researchers. During the interviews, many women described where they came from and how they ended up in the city, and reflected on their experiences of internal migration and urbanisation. Thus, they spoke a range of languages that allowed them to operate in culturally diverse urban contexts and in the process lost some of the technical use of what they identified as their first language.

Furthermore, some health terms translated into the vernacular were not always known and integrated into the daily language of participants. These terms were sometimes better understood in English, or another language, and often it was necessary for researchers too to switch between languages to convey meaning. Many women had attended English secondary schools and were conversant in English even though they did not identify English as a first language. An isiXhosa respondent asked to see the consent script in English alongside the one in isiXhosa and responded:

*“Mm most of the time, like English, even if you speak Afrikaans or I speak Xhosa, like English it is like the middle language, almost everybody, I don’t think or else if other people not understand they can, like prefer the nurse to explain … You see how long it is in Xhosa? Yhu-ah-ah … English is short and simple [reading the Xhosa script] ‘Iinkcukacha zakho’ yoh ah-ah, no man [laughs].”* (C_ANC_ADD_02)

However, while some terms were more easily understood in English than in the vernacular analogue, this did not mean that women found it easier to answer the interview questions in English.

Sometimes participants used two or more languages to elaborate on their responses.

**Researcher:** *And how do you express saying ‘again and again’ [routinely] in Sesotho?*

**Participant**: *Gape le gape [again and again]. (ANC_SOT_02)*

In the quote above the woman uses the Setswana term for “again and again” in her response (rather than Sesotho).

#### Power – fear and trust of the health system

Asymmetry in power relations between participants and the health system were most clearly articulated when asked why they would say “yes” or “no” to the questions asked in the consent process. These were articulated as both fear and trust in the health system.

The first level of asymmetrical power relations was expressed as fear, and was often articulated in relation to nurses. Nurses were described as “rude” and patients feared being scolded by nurses: “*But they are becoming rude and stuff like that*” (ANC_ZUL_06). Being agreeable to the nurses was synonymous with not asking questions, thereby minimising conflict and the potential for poor care by nurses. Thus saying “yes” to the consent questions was one strategy used to fulfil the role of the “good patient”. Participants saw asking questions for more clarity related to consent as a sure way to incur the anger of nurses and to create personal embarrassment.

*“Imagine when they say [imitating a nurse shouting] ‘Wah wah wah wah wah’ to answer you [your questions] … You see? … The embarrassment in front of all the people.”* (C_ANC_ADD_02)

Some women also expressed that it was not an option to ask nurses to repeat information:

*“So, it’s not easy to ask her, ‘Where must I go now?’ Once she told you, [you] must focus, while she shouts, ‘Take this phone! Put this there! Take your card’. So you must focus when she’s talking with you. So that she can’t even repeat the thing that she’s talking about. She’s like that.”* (ANC_ENG_07)

Participants sometimes expressed these ideas even when they had had no personal experience of being poorly treated by nurses; this was because of dominant community discourses around nurses and/or having witnessed poor treatment of other patients by nurses.

Women also reflected that clinics have a dominant language and that it is widely understood in the community that particular languages are used in particular clinics. One woman explained that even though she was not isiZulu speaking, she was careful to speak isiZulu at the clinic [rather than English] because she doesn’t want other clinic attendees and staff to say that she is “*boastful”* and “*they would not give you a service. Go back and speak Setswana or IsiZulu”* (C_ANC_TSW_05). Another participant, an isiXhosa speaker, narrated her experience of navigating between languages at the clinic, and struggling to communicate: “* … sometimes because I’m only understanding Xhosa, I’m not used to other languages … I find a person [health care provider] who does not understand then I have to speak English”* (ANC_ENG_01). This also reflects power dynamics at health centres.

The second level of asymmetrical power relations reflected an overt sense of trust in the health system. This supported adherence to clinic visits and advice offered by nurses.

*“Trust must be with me … I must believe in you [health service] and you [health service] must believe in me only.”* (ANC_SOT_04)

Communication by the health system was seen as an act of care for patients, as something that must be beneficial and therefore something to agree to.

*“A nurse can send me a message … So, that thing comes to me as something important … I must agree to it … it means that she cares about me … ”* (ANC_SOT_06)

Adherence and trust in the rules of the health system was a pathway to care and took precedence over asking questions about the details of care they are receiving. The reasons for this were not apparent, and could be genuine trust in the health system because of positive experiences or as an embodied manifestation of trust in health professionals and the inherent power that rests within that.

## Final consent language script

In response to cognitive interview findings, a final consent language script was developed in five South African languages. Table 6 presents the original and final consent script in English, reflecting the changes made as discussed in the results section. Supplementary Tables 1–4 are the finalised scripts for Setswana, isiXhosa, isiZulu, and Sesotho. Final consent language script is intended for implementation during routine antenatal care services in public health facilities in South Africa.

**Table 6.** Original and final versions of the English consent scripts

Original	Final
*Thank you for visiting the clinic. As you may have experienced, we have collected information about you and the baby you will deliver. This includes your age, weight, health, medicines you take, and similar information about your baby. This information is routinely collected in order for the Department of Health to provide you and your baby with health care services. The Department of Health would also like to ask you if we can use this information for other purposes which I will describe to you now. You can choose whether you agree or not for each of these purposes. It is your decision, and your answers will not change the care you receive. For purposes you agree to, we may ask for some additional information including your contact details.*	*Thank you for visiting the clinic. As you may have experienced, we have collected information about you and the baby you will deliver. This includes your age, weight, health, medicines you take, and similar information about your baby. This information is collected every time you visit the clinic for a check-up in order for the Department of Health to provide you and your baby with health care services. The Department of Health would also like to ask you if we can use this information for other purposes which I will describe to you now. You can choose whether you agree or not for each of these purposes. It is your decision, and your answers will not change the care you receive. For purposes you agree to, we may ask for some additional information including your contact details.*
1. **Can the Department of Health send you general health information about pregnancy and child health via mobile phone through text or WhatsApp?** For example, you will receive MomConnect messages about pregnancy and child health for up to 1 year following the birth of your child.	1. Can the Department of Health send you health information about pregnancy and child health through SMS or WhatsApp? This information will be general to all pregnant women and mothers. For example, you will receive MomConnect messages about pregnancy and child health for up to 1 year following the birth of your child.
2. **Can the Department of Health send you specific health information about pregnancy and child health via mobile phone through text or WhatsApp?** For example, you will receive MomConnect messages about pregnancy and child health for up to 1 year following the birth of your child.	2. Can the Department of Health send you specific information about **your** pregnancy and **your** child’s health through SMS or WhatsApp? For example, you may receive alerts and reminders about your clinic visits, test results, and medicines.
3. **Can your health information be used without your name or personal details?** This information will be used to understand and improve the health of mothers and children and the health services they receive.	3. Can your health information be used anonymously (meaning: without your name or personal details) in health research? This information will be used by health researchers to understand and improve health services for mothers and children.
**4. Can your child’s health information [up until their 5th birthday] be used without your name, your child’s name, or personal details?** This information will be used to better understand and improve the health of mothers and children and how health services are delivered to them.	4. Can your child’s health information [up until their 5th birthday] be used **anonymously** (meaning: without your name, your child’s name, or personal details)? This information will be used **by health researchers** to better understand and improve how health services are delivered to mothers and children.
**5. Can the Department of Health contact you by telephone in the future to invite you to join other research studies?** We will keep your phone number and contact you if there are other studies you could take part in. At that time, you can decide whether you wish to participate or not.	5. Can the Department of Health contact you by phone in the future to invite you to join other research? We will keep your phone number and contact you if there is other research you could take part in. At that time, you can decide whether you wish to participate or not.

In Table 7 we provide some recommendations for how to address each barrier. The barriers at the top of the pyramid – the tip of the iceberg – are easier to address than those at the base. We do however offer some ideas for what researchers may consider to address these; these may vary across contexts and would need to be tailored to particular health and/or research settings.

**Table 7.** Recommendations to address the barriers to informed consent

Barrier to informed consent	Recommendations to address this barrier
Inappropriate vocabulary	• Use alternate words, synonyms and examples to improve comprehension. • Consider using a mix of languages in multilingual contexts.
Cognitive mismatches	• Ensure that words like anonymous, research and study are explained fully. • Use cognitive interviews to understand cognitive failures for each domain of the consent script when being used in a new or different setting.
Linguistic gaps	• Assess the population’s linguistic diversity, and develop and test consent forms in multiple languages. • Train frontline workers to ask individuals about their preferred language for consent rather than assume or conflate language and identity.
Power	• Consider using non-health staff i.e. staff with less medical power for informed consent processes to reduce power asymmetries and encourage patients to ask questions. This would need to be assessed in different contexts for its appropriateness and feasibility. • Train nurses/consent staff on informed consent processes including ways to encourage patients to ask questions, and to be able to answer these questions. Nurses/consent staff must be made aware that patients often feel the need to be agreeable to health care staff^18^ and that this must be avoided. • Consider consent “refreshers” or reminders over time so that consent stays contemporary and subsequent consenting refreshers can be opportunities to address any misunderstandings (dynamic consent).

### Discussion

Our study findings reflect the four main barriers to informed consent regarding data use. The first is with inappropriate wording, where words selected to convey the consent information and questions are not well understood by the women being asked to provide consent. These wording issues are the tip of the iceberg and are often easily fixed by using a different word, rephrasing, or providing examples. The second barrier is cognitive mismatch, where the intended concepts in the consent scripts do not match the meanings that respondents ascribe to those concepts. These are not linguistic barriers but are instead mismatches between the way people with differing worldviews think about concepts including anonymisation and research. The third barrier, linguistic gaps, stems from challenges in providing a consent script in a language that the respondent understands. Linguistic gaps in South Africa often arise from a conflation of cultural identity and linguistic identity, and being “between languages”, wherein no one language covers all linguistic requirements but instead communication and comprehension draw from diverse combinations of languages. Finally, at the base of the pyramid are issues of power asymmetry within the health system that can interfere with genuine informed consent. Women may fear being mistreated by health workers if they do not agree to everything they are asked and even if they ask questions to clarify the consent script. On the other hand, women may imbue medical power with inherent trust, resulting in them agreeing to everything they are asked regardless of whether they understand or want it, simply because they assume the health workers would not ask them to do anything bad. In all cases, these barriers result in women refusing consent for a service or opportunity that they may actually want (by answering NO) or providing consent for a service or opportunity that they do not actually want (by answering YES).

Cognitive mismatches and linguistic gaps are reflective of cultural and language identity; these are especially visible in contexts of high rates of internal and cross-border migration. Other studies found similar challenges. For example, a study looking at the consent process for health research with Burmese migrants in Australia found that some of the challenges were specific to the unique values of people born in Burma, such as unfamiliarity with Western research concepts. Participants in this study also preferred to choose their own interpreters.^19^ A systematic review by Halkoaho et al.^20^ found that cultural differences and the ability of researchers to work in culturally sensitive ways are key factors in improving study participation and retention in multicultural contexts. Thus, more attention is needed to ensure that both translations and cross-cultural communications are effective.^21^ Power imbalances within health care provision are well established;^22^ so too is asymmetry in power relations in the context of decision-making and consent.^23^ Drawing on data from a study in Eswatini, Brear et al^24^ highlight the multiple structural influences that impact participation decisions, and argue that the consent interaction is “inherently power-laden”.

Our findings on inappropriate vocabulary and cognitive mismatches echo similar research findings from cognitive interviews conducted in other contexts. Research in Malawi on family planning^25^ found, for instance, that the Chichewa word *zolerera* [contraceptives] was understood narrowly as pills, condoms, and injectables but did not extend to male or female sterilisation. In England, even without any translation, a tool on how parents manage to care for a child with chronic illness was found to need several language adjustments to ensure cognitive match. For example, the authors added text to clarify that the statement, “We have guiding beliefs that help us manage our child’s condition”, did not specifically refer to whether the respondent was religious.^26^ Research in India on respectful maternity care,^11^ family planning and infant and young child feeding^12^ identified a wide range of vocabulary issues as well as complex mismatched cognitive domains. For example, a quality of care question, “Would you return to this health facility for a future delivery?” sought to assess whether respondents had had positive overall experiences at the facility. Some women, however, would reply “no” because they had been surgically sterilised and thus would not be having another delivery, even though they had a positive experience at the facility. Others would reply “yes” because they did not anticipate having enough money to go to a different, private, facility in the future, even though they were not satisfied with the care received at their local public facility.^11^ The similarity of findings across contexts highlights that is it fundamentally unpredictable how vocabulary words will be interpreted by respondents or which cognitive domains will be mismatched. More broadly, the generalisability of these findings builds the evidence base in favour of integrating cognitive interviewing in all survey tool and consent form development.

This study’s findings illustrate that translation is first in a series of steps that need to be undertaken to bolster patient-centred informed consent processes, and challenged some of the basic assumptions of the research as to whether translation into local languages improved cognitive understanding. We learnt that improving informed consent practices takes more than attending to language, and that it is equally important to pay attention to the cultural and communicative aspects of informed consent^20,21,27^ that are deeply embedded in social and cultural contexts (urban/rural, migration, mix of languages, identity, culture). The study location in urban areas illustrated the diversity in South Africa’s urban populations, often evident as multilingual capabilities (p. 464).^28^ The study highlighted that culture and language must be explored and understood as two separate yet intersecting concepts, that result in the fluidity of language in context. A further consideration for improving informed consent processes, particularly for ongoing use of personal data for health research, is that participants think of research as a chance for them to learn, and that researchers should therefore ensure that research meets this expectation as best as possible or explicitly dispel this misunderstanding during consent processes.

### Limitations

Research activities were conducted in two of South Africa’s nine provinces in urban and peri-urban settings among patients attending public sector maternity services. Findings may differ for other regions and contexts in South Africa. Within sites, we note additionally that recruitment varied as a result of COVID-19 infections and related safety protocols in clinics during data collection.

### Conclusions

The study findings are the first of their kind to present consent language script for onward health data use in multiple African languages for use in capturing beneficiary preferences as part of routine antenatal care services in public sector health facilities. We illustrate the value of cognitive interviews as part of the process for developing consent documents and survey questions, given the high possibility of cognitive mismatch and potentially major consequences of asking “bad” questions. We highlight that addressing language barriers to informed consent is only one in a series of barriers that must be addressed. Policy and practice to improve informed consent processes must be adapted to diverse local contexts and recognise the fluidity of language in multilingual and culturally diverse contexts, and attend to existing power dynamics that hamper health service delivery.

## Supplementary Material

Supplemental Tables 1-6
