# Supplementary material for: Translating the consent form is the tip of the iceberg: using cognitive interviews to assess the barriers to informed consent in South African health facilities
Source: Sex Reprod Health Matters. 2024 Jan 26;31(4):2302553. doi: 10.1080/26410397.2024.2302553 (PMC10823893; doi:10.1080/26410397.2024.2302553)
Supplement: Supplemental Tables 1-6 [file ZRHM_A_2302553_SM4707.docx]

**Supplementary Table 1. Final English Consent Script**

| Thank you for visiting the clinic. As you may have experienced, we have collected information about you and the baby you will deliver. This includes your age, weight, health, medicines you take, and similar information about your baby. This information is collected every time you visit the clinic for a check-up in order for the Department of Health to provide you and your baby with health care services. The Department of Health would also like to ask you if we can use this information for other purposes which I will describe to you now. You can choose whether you agree or not for each of these purposes. It is your decision, and your answers will not change the care you receive. For purposes you agree to, we may ask for some additional information including your contact details. | | | |
| --- | --- | --- | --- |
| 1. | Can the Department of Health send you health information about pregnancy and child health through SMS or WhatsApp? This information will be general to all pregnant women and mothers. For example, you will receive MomConnect messages about pregnancy and child health for up to 1 year following the birth of your child. | Yes | No |
| 2. | Can the Department of Health send you specific information about **your** pregnancy and **your** child’s health through SMS or WhatsApp? For example, you may receive alerts and reminders about your clinic visits, test results, and medicines. | Yes | No |
| 3. | Can your health information be used anonymously (meaning: without your name or personal details) in health research? This information will be used by health researchers to understand and improve health services for mothers and children. | Yes | No |
| 4. | Can your child’s health information [up until their 5th birthday] be used **anonymously** (meaning: without your name, your child’s name, or personal details)? This information will be used **by health researchers** to better understand and improve how health services are delivered to mothers and children. | Yes | No |
| 5. | Can the Department of Health contact you by phone in the future to invite you to join other research? We will keep your phone number and contact you if there is other research you could take part in. At that time, you can decide whether you wish to participate or not. | Yes | No |

**Supplementary Table 2. Final SeSotho Consent Script**

| Rea ho amohela kliniking ya rona. Joalo ka ha o se o hlokometse, re kopantse tsebo ea bophelo ba hau le ngoana eo u tlang ho mmeleha. Sena se kenyelletsa lilemo tsa hau, boima ba mele wa hau, bophelo, meriana eo u e nkang, le ditlhaiso-leseli tsebo tse e tswoanang ka ngoana oa hau. Tlhahisoleseling ena e bokelloa ka mehla le mehla ha o re etela mona kliniking, molemong oa hore Lefapha la Bophelo le fe uena le ngoana wa hau litšebeletso tsa tlhokomelo ea bophelo. Lefapha la Bophelo le boetse le rata ho u botsa hore na re ka sebelisa tlhahisoleseling ena molemong oa tse ling tseo ke tla u hlalosetsa tsona hona joale. O ka khetha hore na o lumela kapa che bakeng sa merero ena. Ke qeto ea hau, mme likarabo tsa hau ha di na ho fetola thuso eo u e fumanang. Bakeng sa merero eo u lumellanang le eona, re ka kopa tlhaiso-leseling e tlatselletsang ho kenyeletsoa lintlha tsa hau tsa puisano. | | | |
| --- | --- | --- | --- |
| 1. | **Na Lefapha la Bophelo le ka u romella tlhaiso-leseling e akaretsang ea bophelo ka boimana le bophelo ba ngoana ka molaetsa wa sms kapa WhatsApp?** Mohlala, o tla amohela melaetsa ea MomConnect ka boimana le bophelo ba ngoana oa hao ho fihlela selemo se le seng kamora ho hlaha ha ngoana oa hau. | Eeh | Che |
| 2. | **Na Lefapha la Bophelo le ka u romella melaetsa ka boimana ba hau le bophelo ba ngoana oa hau mohaleng ka molaetsa wa sms kapa WhatsApp?** Mohlala, o ka fumana litlhokomeliso le likgopotso ka ketelo ea hau ea tleliniki, liphetho tsa liteko le meriana. | Eeh | Che |
| 3. | **Na Tlhaiso-leseding ea hau ea bophelo e ka sebelisoa ntle le lebitso la hau kapa lintlha tsa hau?** Tlhaiso-leseling ena e tla sebelisoa ho utloisisa bophelo ba bo-mme le bana le ho ntlafatsa le litsebeletso tsa bophelo tse ba li fumanang. | Eeh | Che |
| 4. | **Na tlhahisoleseling ea ngoana oa hau ea bophelo [ho fihlela a le lilemo li 5] e ka sebelisoa ntle le lebitso la hau, lebitso la ngoana oa hau kapa lintlha tsa hau?** Tlhaiso-leseling ena e tla sebelisoa ho utloisisa le ho ntlafatsa bophelo ba bo-mme le bana le hore na litšebeletso tsa bophelo li fuoa joang ho bona. | Eeh | Che |
| 5. | **Na Lefapha la Bophelo le ka ikopanya le uena ka mohala nakong e tlang ho u mema hore u kenele lithuto tse ling tsa lipatlisiso?** Re tla boloka nomoro ea hau ea mohala mme re ikopanye le uena haeba ho na le lithuto tse ling tseo u ka nkang karolo ho tsona.Ka nako eo, o ka nka qeto ea hore na o lakatsa ho nka karolo kapa che. | Eeh | Che |

**Supplementary Table 3. Final SeTswana Consent Script**

| Ke lebogela gore o be o etetse tliliniki ya rona. Jaaka ga o kane o kile wa itemogela, re kokoantse tshedimosetso ka wena le ngwana o tlileng go mo tshola. E akaretsa dingwaga tsa gago, boima jwa mmele, ditlhare tse o di dirisang, ga mmogo le yona tshedimosetso eo ka ngwana wa gago. Tshedimosetso e kokoanngwa kgapetsa-kgapetsa go thusa Lefapha la Pholo go neela ditirelo tsa pholo tse di tshwanelang wena le ngwana wa gago. Lefapha la Pholo le eletsa gore o re fe tetla go dirisa tshedimosetso e mabapi le ditiriso tse dingwe tse ke tla go bolelelang ka tsona. O tla itlhophela gore ke ditiriso dife tse o dumalanang le tsona, gongwe tse o sa dumalaneng le tsona. Ke tshwetso ya gago, e bile dikarabo tsa gago di ka se fetole tlhokomelo e o tla e amogelang. Go ya ka ditiriso tse o tla di dumelang, re ka nna ra kopa tshedimosetso e nngwe gape ga mmogo le ya dintlha tsa gago tsa ikgolaganyo. | | | |
| --- | --- | --- | --- |
| 1. | **A Lefapha la Pholo le ka go romelela tshedimosetso kakaretso ya pholo ka boimana jwa gago le pholo ya ngwana ka mogala wa gago wa letheka ka molaetsa-khutswe (SMS) gongwe WhatsApp?** Sekai - o tla amogela melaetsa ya MomConnect mabapi le boimana le pholo ya ngwana go fitlhelela a tshwara ngwaga. | Ee | Nyaa |
| 2. | **A Lefapha la Pholo le ka go romelela tshedimosetso ka boimana jwa gago le pholo ya ngwana wa gago ka mogala wa gago wa letheka ka molaetsa-khutswe (SMS) gongwe WhatsApp?** Sekai, o ka amogela melaetsa ga go go gopotsa matsatsi a go etela tliliniki, dipholo tsa ditlhatlhobo le ditlhare. | Ee | Nyaa |
| 3. | **A tshedimosetso ya gago ya pholo e ka dirisiwa ntle le leina gongwe kitso ya namana ya gago?** Tshedimosetso e tla dirisiwa go tlhaloganya le go tokafatsa pholo ya basadi le bana, ga mmogo le ditirelo tse ba di amogelang. | Ee | Nyaa |
| 4. | **A tshedimosetso ya ngwana wa gago ya pholo [go fitlhela a le dingwaga tse 5 (tlaanu)] e ka dirisiwa ntle le leina la gago, leina la ngwana wa gago, gongwe kitso ya namana ya gago?** Tshedimosetso e tla dirisiwa go tlhaloganya le go tokafatsa pholo ya basadi le bana, ga mmogo le gore ditirelo tse ba di amogelang di neelwa jang. | Ee | Nyaa |
| 5. | **A Lefapha la Pholo le ka go founela mo isagong go go laletsa go tsenela dipatlisiso tsa pholo?** Re tla boloka dinnomoro tsa gago tsa mogala re be re go founela fa go na le dipatlisiso tse dingwe tse o ka di tsenelang. Ka nako eo, o tla tsaya tshwetso ya gore a o eletsa go tsaya karolo gongwe nnyaa. | Ee | Nyaa |

**Supplementary Table 4. Final IsiXhosa Consent Script**

| Enkosi ngotyelela iklinikhi. Njengokuba ubonile, siqokelele iinkcukacha ezimalunga nawe nosana lwakho ozakulibeleka. Oku kuquka ubudala, ubunzima bomzimba, isimo sempilo, amayeza owathathayo kwakunye neenkcukacha ezibunjalo ngomntwana wakho. Ezinkcukacha ziqokelelwa rhoqo ukuze isebe lezempilo linike wena nomntwana wakho iinkonzo zempilo ezikufaneleyo. Isebe lezempilo lingathanda ukucela ukuba lisebenzise ezi nkcukacha malunga neenjongo endizakuthi ndikuxelele ngazo ngoku. Ungakhetha ukuba uyavuma okanye uyala kwinjongo nganye. Sisigqibo sakho kwaye iimpendulo zakho azisayikutshintsha inkathalelo oyifumanayo. Malunga neenjongo ovumelana nazo, kungenzeka sicele iinkcukacha ezongezelelekileyo kuquka neenombolo zakho. Ndizokunika inombolo nenckukacha ongazithatha uyenazo ekhaya. | | | |
| --- | --- | --- | --- |
| 1. | **Isebe lezempilo lingakuthumelela iinkcukacha (i-information) zempilo ezithe gabalala/ezibanzi malunga nokukhulelwa kwakunye nesimo sempilo yomntwana ngomnxeba ngokuthi uthunyelelwe iSMS okanye uWhatsApp?** Umzekelo, uzakufumana iiSMS ezisuka kuMomConnect malunga nokukhulelwa kwanesimo sempilo yomntwana wakho kangangonyaka emva kokuba umntwana wakho ezelwe. | Ewe | Hayi |
| 2. | **Ingaba isebe lezempilo okanye Umnyango Wezempilo lingakuthumelela iinkukacha malunga nokukhulelwa kwakho kwanesimo sempilo yomntwana wakho lisebenzisa ifowuni okanye uWhatsApp?** Umzekelo, ungafumana izilumkiso nemiyalezo ekukhumbuzayo malunga notyelela kwakho eklinikhi, iziphumo zovavanyo namayeza. | Ewe | Hayi |
| 3. | **Ingaba iinkcukacha zakho zempilo zingasetyenziswa ngaphandle kwegama lakho?** Ezi nkcukacha zizakusetyenziswa ukuqonda kwanokuphucula impilo yoomama nabantwana kwaneenkonzo zempilo abazifumanayo. | Ewe | Hayi |
| 4. | **Ingaba iinkcukacha ezinge simo sempilo yomntwana wakho (de ayofikelela kumnyaka wesihlanu ezelwe) zingasetyenziswa na ngaphandle kwegama lakho, igama lakhe okanye iinkcukacha zenu?** Ezinkcukacha zizakusetyenziselwa ukuqonda nokuphucula impilo yoomama nabantwana kwanendlela abanikezwa ngazo iinkonzo zempilo. | Ewe | Hayi |
| 5. | **Ingaba isebe lezempilo lingakutsalela umnxeba kwixesha elizayo likumema ukuba ubeyinxalenye yolunye uphando?** Sizakugcina iinombolo zakho ze sinxulumane nawe ukuba kukho olunye uphando onokuthi uthathe inxaxheba kulo. Ngeloxesha ungathatha isigqibo sokuba uyanqwenela okanye awunqweneli ukuthatha inxaxheba | Ewe | Hayi |

**Supplementary Table 5. Final IsiZulu Consent Script**

| Siyabonga ngokuvakashela umtholampilo. Kungenzeka ukuthi usuhlangabezane nakho, sesiqoqe imininingwane ngawe nengane ozoyibeletha. Lokhu kubandakanya iminyaka yakho yokuzalwa, isisindo sakho, isimo sempilo, imithi oyithathayo, kanye nemininingwane efanayo mayelana nengane yakho. Lemininingwane iqoqwa njalo ukwelekelela uMnyango Wezempilo ukuze unike wena kanye nengane yakho ngezinsizakalo ezifanele. UMnyango Wezempilo ungathanda ukwazi ukuthi singa yisebenzisa yini imininingwane yakho noma eyengane yakho kwezinye izinhloso engizokuchazela ngazo manje. Ungakhetha ukuthi uyavuma noma cha. Kuyisinqumo sakho, futhi izimpendulo zakho ngeke zishintshe ukunakekelwa ozokuthola. Uma uvuma, singacela imininingwane eyengeziwe kanye nendlela esingaxhumana nawe ngayo. | | | |
| --- | --- | --- | --- |
| 1. | **Ingabe uMnyango Wezempilo ungakuthumela ulwazi olujwayelekile mayelana nokukhulelwa kwabesifazane nempilo yengane, ngeSMS noma ngeWhatsApp?** Isibonelo, uzothola imilayezo ye-MomConnect mayelana nokukhulelwa nempilo yengane kuze kube unyaka owodwa ngemuva kokuzalwa kwengane yakho. | Yebo | Cha |
| 2. | **Ingabe uMnyango Wezempilo ungakuthumela ulwazi oluthile mayelana nokukhulelwa kwakho kanye nempilo yengane yakho ngeSMS noma ngeWhatsApp?** Isibonelo, ungathola izexwayiso nezikhumbuzo ngokuvakasha kwakho emtholampilo, imiphumela yokuhlolwa kanye nemithi oyithathayo. | Yebo | Cha |
| 3. | **Ingabe imininingwane yakho yempilo ingasetshenziswa ezifundweni zocwaningo ngaphandle kwegama lakho noma imininingwane ekhombisa wena?** Lolu lwazi luzosetshenziselwa ukuqonda kabanzi nokuthuthukisa impilo yomama nezingane kanye nokunakekelwa abakutholayo. | Yebo | Cha |
| 4. | **Ingabe imininingwane yempilo yengane yakho [kuze kufike unyaka wayo wokuzalwa wesi-hlanu] ingasetshenziswa ezifundweni zocwaningo ngaphandle kwegama lakho, igama lengane yakho, noma imininingwane ekhombisa wena?** Lolu lwazi luzosetshenziselwa ukwazi kabanzi nokuthuthukisa isimo sempilo yomama kanye nezingane, nokuthi izinsizakalo zezempilo zilethwa kanjani kubo. | Yebo | Cha |
| 5. | **Ingabe uMnyango Wezempilo ungaxhumana nawe ngocingo ngesikhathi esizayo ukumeme ukuthi ube yingxenye yezinye izifundo zocwaningo?** Sizogcina inombolo yakho yocingo futhi sixhumane nawe uma kukhona ezinye izinhlelo ongabamba iqhaza kuzo. Ngaleso sikhathi, unganquma ukuthi uyafuna yini ukubamba iqhaza kulezo zinhlelo noma cha. | Yebo | Cha |

**Supplementary Table 6. Afrikaans Consent Script Version 1**

| Dankie vir jou (U) kliniek besoek vandag. Soos u dalk ervaar het, het ons inligting oor u en die baba wat u sal lewer, versamel. Dit sluit in jou (u) ouderdom, gewig, gesondheid, medikasie wat u neem, en soortgelyke inligting oor u baba. Hierdie inligting word gereeld ingesamel sodat die Departement van Gesondheid dienste aan jou en jou baba kan voorsien. Die Departement van Gesondheid wil ook vra of ons hierdie inligting vir ander doeleindes kan gebruik. Ek sal hierdie doeleindes nou aan u verduidelik. Jy kan besluit of jy toestemming vir elkeen van dié doeleindes will gee of nie. Dit is jou besluit, en jou antwoorde sal geen verskil maak aan die sorg wat jy ontvang nie. Vir doeleindes waartoe u instem, mag ons addisionele inligting vra, insluitend jou kontakbesonderhede. | | | |
| --- | --- | --- | --- |
| 1. | **Kan die Departement van Gesondheid algemene gesondheidsinligting oor swangerskap en kindergesondheid deur middel van teks of Whatsapp boodskappe na jou selfoon stuur?** Byvoorbeeld, jy/u sal MomConnect-boodskappe oor swangerskap en kindergesondheid vir tot 1 jaar na die geboorte van jy/u kind ontvang. | Ja | Nee |
| 2. | **Kan die Departement van Gesondheid vir jou inligting stuur oor jou swangerskap en jou kind se gesondheid deur middel van teks of Whatsapp boodskappe na jou selfoon stuur?** Byvoorbeeld, jy/u mag kennisgewings en herinneringe oor u kliniekbesoeke, toets uitslae en medisyne ontvang . | Ja | Nee |
| 3. | **Mag jou gesondheidsinligting sonder jou naam of persoonlike besonderhede gebruik word?** Hierdie inligting sal gebruik word om die gesondheid van moeders en kinders en die gesondheidsdienste wat hulle ontvang, te verstaan en te verbeter. | Ja | Nee |
| 4. | **Mag jou/u kind se gesondheidsinligting [tot hul 5de verjaarsdag] gebruik word sonderom jou/u naam, jou/u kind se naam, of persoonlike besonderhede te noem?** Die gebruik van hierdie inligting sal ons help om die gesondheid en dienslewering aan moeders en kinders te verstaan en vebeter. | Ja | Nee |
| 5. | **Mag die Departement van Gesondheid jou in die toekoms telefonies kontak om vir jou te nooi om by ander navorsingstudies aan te sluit?** Ons sal jou telefoonnommer hou en jou kontak as daar ander studies is waaraan jy kan deelneem. Op daardie stadium kan jy besluit of jy wil deelneem of nie. | Ja | Nee |
